# Structural characterization of the native oligomerization mode of MvaT proteins in *Pseudomonas*

**DOI:** 10.1128/spectrum.00235-26

**Published:** 2026-04-06

**Authors:** Delyana Vasileva, Chiho Suzuki-Minakuchi, Takatoshi Arakawa, Yoshitaka Moriwaki, Kento Yonezawa, Nobutaka Shimizu, Zui Fujimoto, Tohru Terada, Kazunori Okada, Hideaki Nojiri

**Affiliations:** 1 Agro-Biotechnology Research Center, Graduate School of Agricultural and Life Sciences, The University of Tokyo 515734 , Tokyo, Japan; 2 Collaborative Research Institute for Innovative Microbiology, The University of Tokyo 515734 , Tokyo, Japan; 3 Department of Biotechnology, Graduate School of Agricultural and Life Sciences, The University of Tokyo 515734 , Tokyo, Japan; 4 Faculty of Pharmaceutical Sciences, Tokyo University of Science, Chiba, Japan; 5 Medical Research Laboratory, Institute of Integrated Research, Institute of Science Tokyo, Tokyo, Japan; 6 Institute of Materials Structure Science, High Energy Accelerator Research Organization (KEK), Ibaraki, Japan; 7 Center for Digital Green-innovation, Nara Institute of Science and Technology, Nara, Japan; 8 Life Science Research Infrastructure Group, R&D of Technology and Systems for Synchrotron Radiation Applications Division, RIKEN SPring-8 Center, Hyogo, Japan; 9 Research Center for Advanced Analysis, National Agriculture and Food Research Organization (NARO), Ibaraki, Japan; Connecticut Agricultural Experiment Station, New Haven, Connecticut, USA

**Keywords:** nucleoid-associated proteins, H-NS, *Pseudomonas*, X-ray crystallography, small angle X-ray scattering

## Abstract

Histone-like protein H1 (H-NS) proteins are key regulators of horizontally acquired genes in enterobacteria. Functional analogs of H-NS, collectively known as H-NS family proteins, have been discovered in diverse bacteria and on large transmissible plasmids. These proteins share the ability to form oligomers through their N-terminal oligomerization domain, a feature critical for their regulatory functions. The N-terminal domain of MvaT proteins, members of the H-NS family proteins in pseudomonads, consists of a central and a terminal dimerization site. We previously elucidated the structure of the central dimerization site of TurB, an MvaT homolog in *P. putida* KT2440. Here, we solved the crystal and solution structures of its terminal dimerization site and generated a structural model of TurB in an oligomerized form. Our data show that the terminal dimerization site of TurB is composed of two helices connected by a flexible hinge. The results also reveal differences between the oligomerization manner of MvaT proteins and H-NS in enterobacteria. Residues forming salt bridges and hydrophobic interactions that stabilize the terminal dimerization site are relatively conserved among MvaT proteins encoded on the chromosomes of various *Pseudomonas* strains and plasmids. Previous studies have shown that *Pseudomonas* strains typically harbor plasmids encoding MvaT proteins, while enterobacteria carry plasmids encoding H-NS. Our results suggest that differences in the oligomerization domain of H-NS family proteins may be critical for the cross-talk between plasmids and the host chromosome.

**IMPORTANCE:**

Horizontal gene transfer is a major driver of microbial evolution. H-NS family proteins are key factors in optimizing the transcription of newly acquired genes in host cells. Despite high sequence diversity among bacteria, these proteins share the ability to form oligomers along DNA, a critical property for their function. H-NS family proteins encoded both on large transmissible plasmids and on the chromosomes of host cells play an important role in the successful integration of new genetic sequences into the regulatory networks of microbes. Bacterial hosts typically harbor plasmids encoding the same type of H-NS family proteins as found on their chromosomes. Here, we characterized the structural basis for oligomerization of MvaT proteins, members of the H-NS family proteins in pseudomonads. Our findings reveal differences between the oligomerization mode of MvaT proteins and H-NS in enterobacteria, suggesting that these differences might impact the transmission routes of plasmids within microbial communities.

## INTRODUCTION

Nucleoid-associated proteins (NAPs) are abundant architectural proteins that play a fundamental role in maintaining an organized state of the bacterial chromosome and act as global transcriptional regulators (1, 2). H-NS family proteins, one of the best-characterized NAPs, repress the expression of genes acquired through horizontal gene transfer by preferentially binding to AT-rich genomic regions, a process termed xenogeneic silencing (3–5). Xenogeneic silencing is thought to mitigate any potential fitness cost due to uncontrolled expression of foreign genes and, at the same time, facilitates integration of new genetic material into the regulatory networks of the host cells (6, 7). Although initial studies focused on H-NS homologs in enterobacteria, further analyses revealed that functional analogs of H-NS exist in both gram-negative and -positive bacteria. Members of the H-NS family proteins include H-NS of enterobacteria, Lsr2 of *Actinomycetes*, MvaT of *Pseudomonas*, and Rok of *Bacillus* species (8). H-NS family proteins are also encoded on mobile genetic elements (MGE), such as plasmids and genomic islands (9–11). Previous biochemical and transcriptomic studies have demonstrated that chromosome- and plasmid-encoded H-NS family proteins interact with each other and cooperatively regulate a number of genes in host cells, which is key for the successful accommodation of newly acquired DNA (12–15).

Despite low sequence similarity, H-NS family proteins share a common structural architecture, consisting of an N-terminal oligomerization domain and a C-terminal DNA-binding domain joined by a linker region (8). The ability of H-NS family proteins to form high-order oligomeric structures is critical for their function as DNA-structuring proteins and transcriptional regulators (16–18). Formation of oligomeric filaments along DNA is achieved by two dimerization sites located in the N-terminal domain. The N-terminal oligomerization domains have been experimentally determined in H-NS, MvaT, and Lsr2 (19–24).

We previously characterized the oligomerization domain of Pmr, an MvaT homolog encoded on the incompatibility group P-7 (IncP-7) carbazole degradative plasmid pCAR1, which is transmissible among various *Pseudomonas* strains, and identified seven residues important for homo-oligomerization (13). *P. putida* KT2440, a model host for pCAR1, has five genes encoding MvaT proteins (*turA-E*), among which TurA and TurB are expressed predominantly (12). We solved the crystal structure of the N-terminal domain of TurB, which contains two dimerization sites: the terminal dimerization site and the central dimerization site (24). The overall architecture of the central dimerization site was similar to that of enterobacterial H-NS, whereas the terminal dimerization sites of TurB and H-NS appeared to differ. In that study, we used an oligomerization-deficient variant of the N-terminal domain of TurB (TurB_nt_61_-R8A), where R8 in the terminal dimerization site was substituted by alanine. Therefore, the terminal dimerization site in TurB_nt_61_-R8A might have a different structure compared to that in the native protein.

In this study, to obtain a more precise picture of the oligomerization mechanism of MvaT proteins, we determined the crystal and solution structures of the N-terminal domain of TurB containing the native terminal dimerization site. We used a TurB variant, TurB_nt_50_, which contains residues 1–50 and is truncated at the central dimerization site. Our previous biochemical analyses showed that TurB_nt_50_ forms dimers in solution, indicating that it has lost its ability to self-associate via the central dimerization site while retaining an intact terminal dimerization site (24). Using the structures of TurB_nt_61_-R8A and TurB_nt_50_, we generated a structural model for the oligomerization of MvaT proteins.

## RESULTS AND DISCUSSION

### Overall structure of TurB_nt_50_

**Fig 1:**
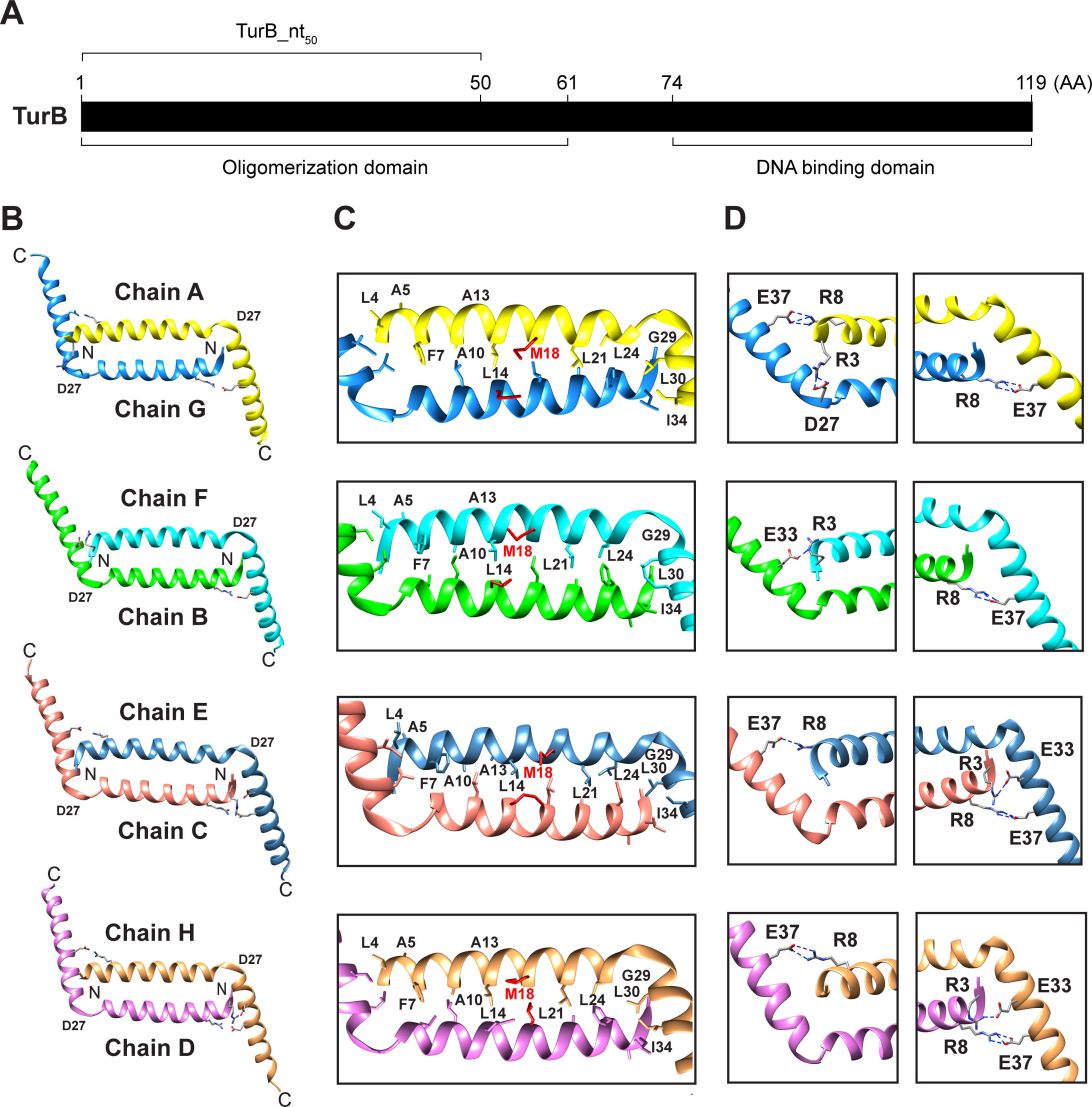
Crystal structure of TurB_nt_50_. (**A**) Schematic representation of the full-length TurB and the region included in the truncated variant TurB_nt_50_. (**B**) A ribbon representation of the overall structure. The eight molecules (Chains A [yellow], B [green], C [salmon], D [pink], E [dark blue], F [cyan], G [blue], and H [brown]) forming four dimers present in the asymmetric unit are shown. The N- and C-termini of all monomers are designated by N and C, respectively. Side chains of the residues participating in the formation of salt bridges are colored by element (gray, carbon atom; blue, nitrogen atom; and red, oxygen atom). (**C**) Side chains of the amino acids involved in hydrophobic interactions are shown. The numbers of these amino acid residues in chains A, F, E, and H are indicated. The side chain of residue M18 is highlighted in red. (**D**) Zoomed-in view of the residues involved in the formation of salt bridges. Salt bridges are indicated by dashed blue lines.

The truncated variant TurB_nt_50_ (Fig. 1A) was purified from *Escherichia coli* BL21 (DE3) and crystallized in a solution containing PEG 3350 as precipitant. The structure was determined *de novo* using the sulfur-based native single-wavelength anomalous dispersion method (S-SAD). For phasing, we used the S-SAD data without molecular replacement searches, as the space groups and unit cell dimensions were the same for both the native crystal and the S-SAD crystal. We then refined the initial TurB_nt_50_ model to 2.7 Å resolution. The refined model produced a crystallographic R-factor of 24.7% and an *R*_free_ value of 28.5%. X-ray diffraction data collection and refinement statistics are summarized in Table 1. The TurB_nt_50_ crystal contained eight protein molecules per asymmetric unit forming four biologically relevant dimers composed of Chain A (amino acid residues 2–52) and Chain G (residues 2–50), Chain F (residues 2–52) and Chain B (residues 2–52), Chain C (residues 2–52) and Chain E (residues 2–52), and Chain D (residues 2–52) and Chain H (residues 2–50), respectively (Fig. 1B; Fig. S1). Residues L51 and E52 arose from the XhoI restriction site in the expression plasmid. Superimposition between Chain A and the other seven chains suggested that the overall structures of all monomers closely resemble each other (root mean square deviation [RMSD] 0.926–2.645 Å for common Cα atomic pairs). Our previous structural study showed that the N-terminal region of TurB_nt_61_-R8A (residues 4–46 in Chain A and residues 5–47 in Chain B) forms one elongated α-helix (24). Here, we demonstrated that each monomer in the TurB_nt_50_ crystal contains two helices with a bend at residue D27 (Fig. 1B).

**TABLE 1:** X-ray diffraction data collection and structure refinement statistics

	TurB_nt_50_	TurB_nt_50_ S-SAD
Diffraction data		
Beamline	PF AR-NE3A	PF BL-1A
Wavelength (Å)	1.0000	2.7000
Resolution range	46.6–2.7 (2.8–2.7)^*a*^	137–3.0 (3.2–3.0)
Space group	P 21 21 21	P 21 21 21
Unit cell	45.69 63.30 275.87 90 90 90	44.8 64.2 274.4 90 90 90
Total reflections	301,394 (40,947)	597,732 (58,912)
Unique reflections	22,936 (2,231)	16,912 (2,447)
Multiplicity	13.1 (13.8)	20.0 (12.8)
Completeness (%)	98.9 (98.8)	96.2 (92.4)
Mean I/sigma(I)	10.9 (2.4)	21.1 (5.5)
Wilson B-factor	59.50	46.92
R-merge	0.140 (1.355)	0.144 (0.471)
R-meas	0.146 (1.407)	0.146 (0.481)
R-pim	0.041 (0.375)	0.024 (0.095)
CC1/2	95.6	99.6
Refinement		
Resolution range (in refinement)	40.9–2.7 (2.8–2.7)	
Reflections used in refinement	22,707 (2,208)	
Reflections used for R-free	2,271 (234)	
R-work	0.247 (0.417)	
R-free	0.285 (0.512)	
Number of non-hydrogen atoms	3,255	
Macromolecules	3,242	
Ligands	0	
Solvent	13	
Protein residues	404	
RMS (bond)	0.009	
RMS (angles)	1.08	
Ramachandran favored (%)	98.2	
Ramachandran allowed (%)	1.55	
Ramachandran outliers (%)	0.26	
Rotamer outliers (%)	3.63	
Clashscore	10.6	
Average B-factor	78.1	
Macromolecules	78.1	
Solvent	63.7	

^*a*^ Statistics for the highest-resolution shell are shown in parenthesis.

### Structural basis for oligomerization of TurB via the terminal dimerization site

**Fig 2:**
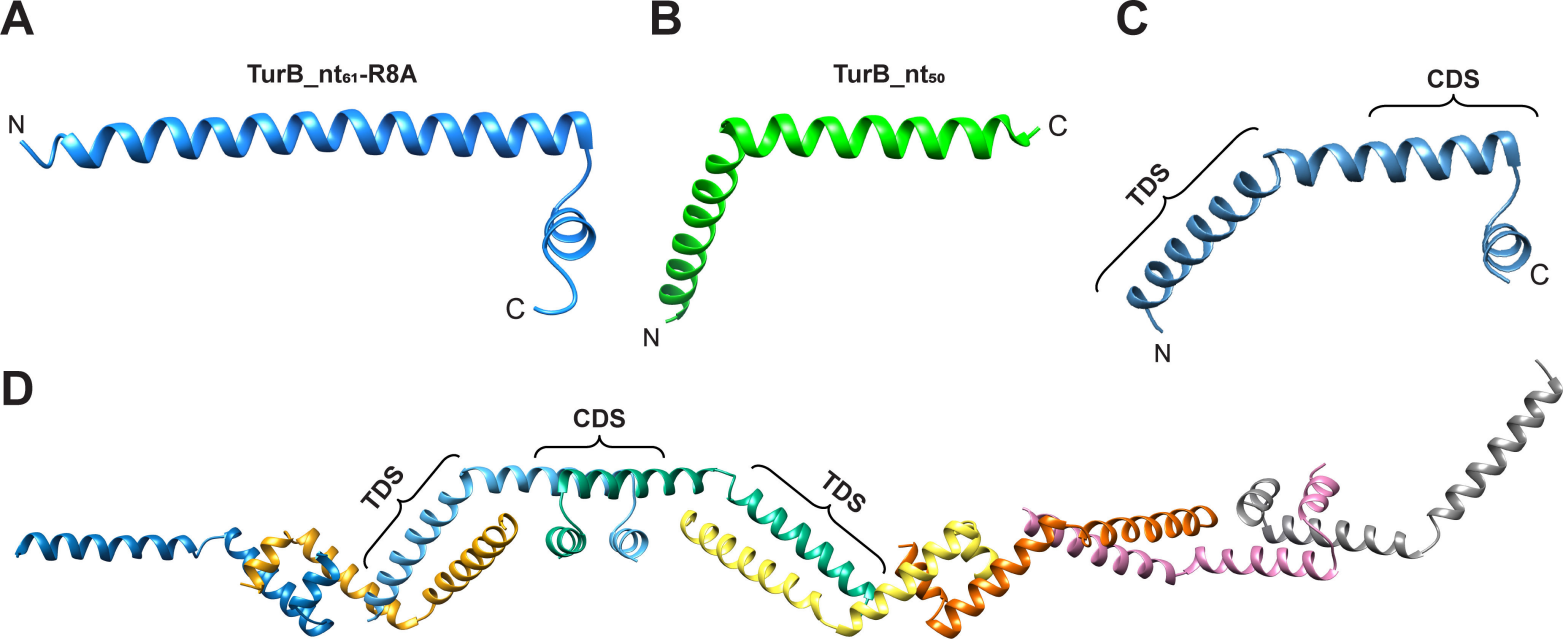
Structural basis for TurB oligomer formation. Structures of (**A**) TurB_nt_61_-R8A (24) and (**B**) TurB_nt_50_ monomers. The N- and C-termini are designed by `N` and `C`. (**C**) A structural model of the native N-terminal oligomerization domain of TurB. The central dimerization site (CDS, residues 32–58), and the terminal dimerization site (TDS, residues 1–31) are indicated. (**D**) An oligomer model of the N-terminal domain of TurB. The model is composed of four dimers connected through their terminal dimerization sites. The individual monomers are highlighted in different colors. CDS and TDS are shown for one of the dimers.

The N-terminal helices of two monomers in each TurB_nt_50_ dimer formed an anti-parallel coiled-coil (Fig. 1B). The interface at this region (residues 2–37) covered an area of 910.7–941.8 Å^2^ and was recognized as a stable dimer by PISA (Chain A-Chain G, ΔG^int^ −15.0 kcal/mol; Chain B- Chain F, ΔG^int^ −14.9 kcal/mol; Chain C- Chain E, ΔG^int^ −15.5 kcal/mol; and Chain D- Chain H, ΔG^int^ −16.3 kcal/mol). Further analysis of the TurB_nt_50_ structure revealed that the terminal dimerization site is stabilized by hydrophobic interactions formed by residues L4, A5, F7, A10, A13, L14, M18, L21, L24, G29, L30, and I34 (Fig. 1C). In addition, salt bridges between the following residues contributed to the stabilization of the terminal dimerization site: R8 (Chain A) and E37 (Chain G); R3 (A) and D27 (G); R8 (G) and E37 (A); R3 (F) and E33 (B); R8 (B) and E37 (F); R3 (C) and E33 (E); R8 (C) and E37 (E); R8 (E) and E37 (C); R3 (D) and E33 (H); R8 (D) and E37 (H); and R8 (H) and E37 (D) (Fig. 1D). These results indicated that substitution of R for A at position eight in the oligomerization-deficient variant TurB_nt_61_-R8A prevents the formation of a salt bridge with E37, resulting in destabilization of the bend at residue D27 and formation of one long α-helix. These findings could also explain our previous biochemical analyses, which showed that M18A and D27A substitutions significantly decrease the homo-oligomerization capacity of TurB_nt_61_ (24). Using the structures of TurB_nt_61_-R8A (Fig. 2A) and TurB_nt_50_ (Fig. 2B) containing intact central and terminal dimerization sites, respectively, we generated models of the native TurB oligomerization domain in monomeric (residues 1–58) (Fig. 2C) and oligomerized state (Fig. 2D). Movie S1 presents the oligomerization model as in Fig. 2D, incorporating the C-terminal DNA-binding domain. The crystal structure of the N-terminal oligomerization domain of H-NS in enterobacteria exhibits a superhelical structure (Movie S2) (22). In contrast, the oligomerization domain of TurB forms a filamentous structure (Fig. 2D). These structural differences may arise from variations in the chromosomal features of enterobacteria and *Pseudomonas* species, such as the GC content of DNA or composition of NAPs in the cell.

### Solution structure of TurB_nt_50_

**Fig 3:**
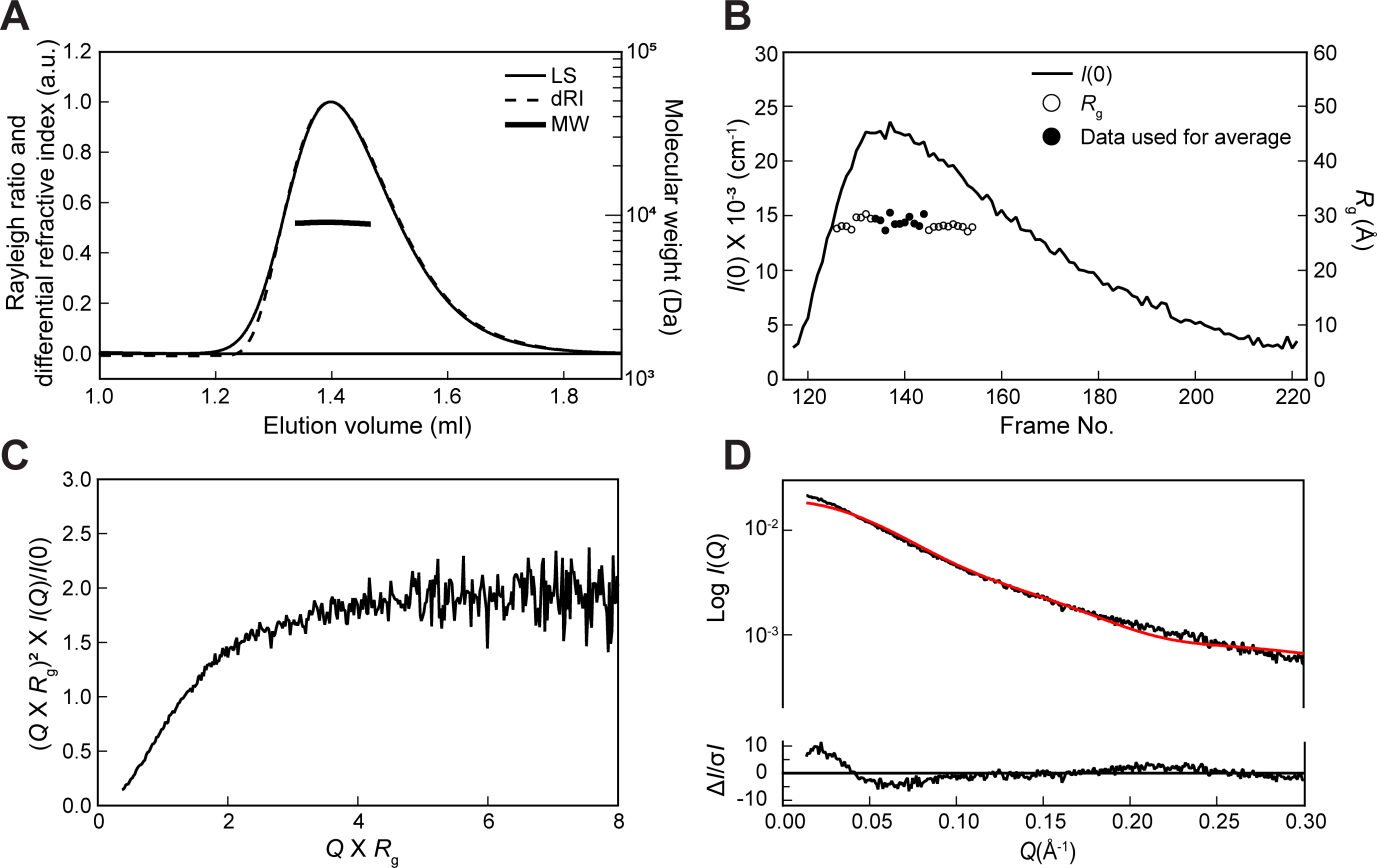
SEC-MALS/RI and SEC-SAXS results of TurB_nt_50_. (**A**) Chromatograms of light scattering (LS) and differential refractive index (dRI) obtained by the SEC-MALS/RI experiment and the molecular weight (MW) distribution obtained from these two chromatograms. (**B**) SEC-SAXS chromatograms of *I*(0) and distribution of *R_g_* values. (**C**) The normalized Kratky plot. (**D**) The theoretical scattering profile calculated from the crystal structure (PDB ID: 8H8H) (red line) was fit to the experimental profile (black line) using CRYSOL. The χ^2^ value is 8.2.

To further evaluate the structural features of TurB_nt_50_, we performed SEC-SAXS analyses. The SEC-MALS/RI and SEC-SAXS chromatogram results showed single peaks (Fig. 3A and B). We detected almost no dispersion of the gyration radius (*R_g_*) near the peak region, which indicates that the scattering profile was obtained from a monodispersed state (Fig. 3B). The molecular weights obtained by SEC-MALS/RI and SEC-SAXS are 8,955 Da and 11,250 Da, respectively. Given that the molecular weight of TurB_nt_50_ calculated from the chemical composition was 5,759 Da, these results suggested that TurB_nt_50_ exists as a dimer in solution. The normalized Kratky plot of the averaged scattering curve near the peak region showed a monotonic increase in scattering intensity toward the wide-angle side with a tendency to plateau at *Q×R_g_* > 4 (Fig. 3C). The theoretical scattering profile calculated from the TurB_nt_50_ crystal structure and the experimental scattering profile were roughly similar but showed some differences (Fig. 3D). In particular, the *R_g_* value of the experimental profile (28.8 ± 1.9 Å) was larger than that of the theoretical profile (24.2 Å), indicating that the N-terminal region of TurB_nt_50_ is in an extended state similar to that in TurB_nt_61_-R8A. The crystal structural data and the SEC-SAXS results suggested that the folding state of TurB_nt_50_ is flexible and it transitions between bent and extended conformation states, which might be important for stable oligomer formation along DNA.

### Conservation of residues important for oligomerization in MvaT homologs

**Fig 4:**
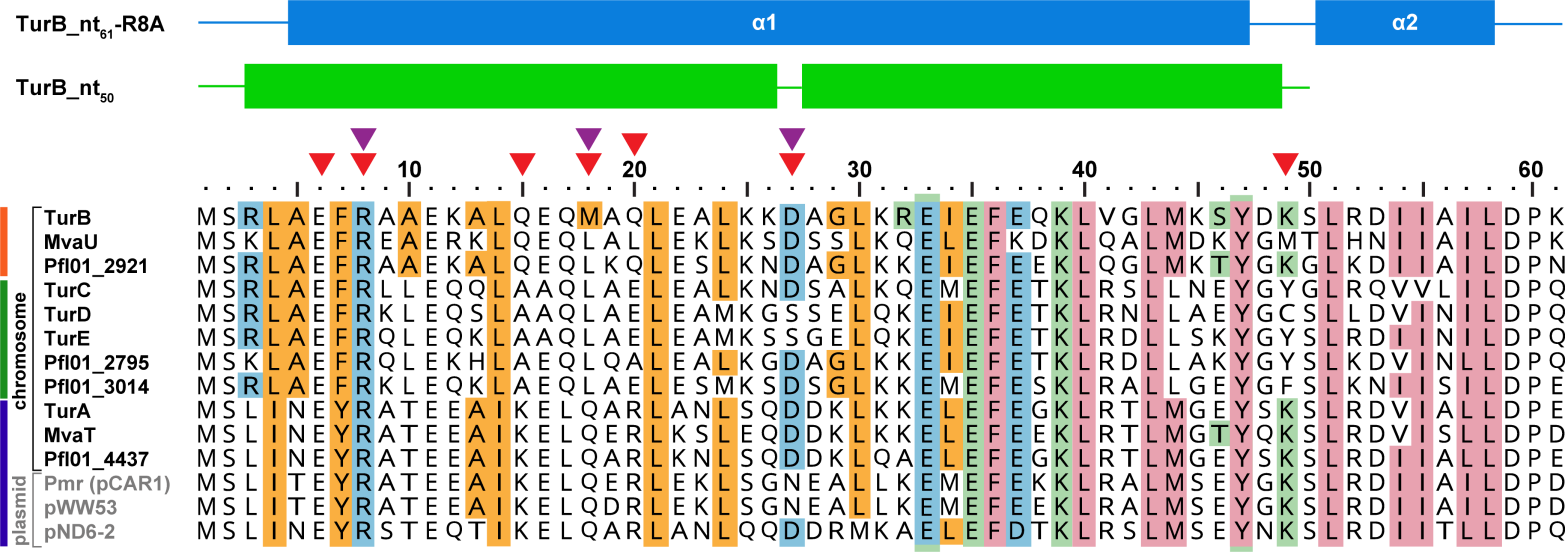
Amino acid sequence alignment of the N-terminal oligomerization domain of MvaT homologs encoded on the chromosomes of different *Pseudomonas* strains and plasmids. Multiple sequence alignment of the N-terminal domains of representative MvaT proteins from Group I, Group II, and Group III (Fig. S2) was performed using CLUSTAL OMEGA software, version 1.2.4 (https://www.ebi.ac.uk/jdispatcher/msa/clustalo). The N-terminal regions (residues 1–61) of TurA (PP_1366), TurB (PP_3765), TurC (PP_0017), TurD (PP_3693), and TurE (PP_2947) of *P. putida* KT2440, MvaT (PA4315), and MvaU (PA2667) of *P. aeruginosa* PAO1, Pfl01_2795, Pfl01_2921, Pfl01_3014, and Pfl01_4437 of *P. fluorescens* Pf0-1, and MvaT proteins encoded on plasmids pCAR1 (Pmr), pWW53, and pND6-2 were used for the alignment. The residues participating in the formation of hydrophobic interactions (orange) and salt bridges (light blue) in the TurB_nt_50_ structure and conserved or similar residues in the other MvaT proteins are highlighted. The residues that form the hydrophobic core and salt bridges stabilizing the central dimerization site in the TurB_nt_61_-R8A structure (24) and the corresponding residues in the other MvaT proteins are shaded in pink and light green, respectively. Residues important for homo-oligomerization of TurB_nt_61_ (24) and Pmr_nt_61_ (13) are indicated by purple and red arrows, respectively. Secondary structural features of TurB_nt_61_-R8A (blue) (24) and TurB_nt_50_ (green) are shown above the sequence alignment; α-helices are indicated as rectangles and unstructured loops as lines. MvaT proteins belonging to Groups I, II, and III (Fig. S2) are indicated by vertical blue, orange, and green lines, respectively.

Bioinformatic analyses revealed that the same types of H-NS family proteins are typically encoded on conjugative plasmids and the chromosomes of their hosts (11). Biochemical and transcriptomic studies showed that the chromosome-encoded TurA and TurB and the pCAR1-encoded Pmr form hetero-oligomers and function cooperatively to facilitate the integration of foreign DNA into the transcriptional networks of host cells (13–15). Similarly, the Sfh, an H-NS ortholog encoded on the plasmid pSfR27, forms heteromeric structures and co-regulates a number of genes with the chromosome-encoded H-NS and its paralog StpA in *Shigella flexneri* 2a strain 2457T (25, 26). Previous phylogenetic analyses have divided the MvaT proteins into three distinct groups (27). Consistent with these studies, our own analysis also identified the same three groups (Fig. S2). The residues stabilizing the terminal dimerization site in the TurB_nt_50_ structure through formation of hydrophobic interactions and salt bridges are relatively conserved in MvaT proteins from Groups I, II, and III encoded on various plasmids and the chromosomes of diverse *Pseudomonas* strains (Fig. 4 and Fig. S2). Previously, we have demonstrated that R8A, Q18A, and N27A substitutions significantly affect the homo-oligomerization ability of the N-terminal oligomerization domain of Pmr (13). In addition, TurB_nt_50_-R8A has a reduced capacity to form heteromeric complexes with TurA and Pmr (15). Collectively, these findings indicate that plasmid- and chromosome-encoded MvaT proteins form homo- and hetero-oligomers in a relatively similar fashion, although non-conserved residues can affect the formation of hetero-oligomers among the MvaT homologs. Given that MvaT, H-NS, and Lsr2 homologs exhibit unique oligomerization manners, we hypothesize that structural variability in the N-terminal oligomerization domain of H-NS family proteins plays an important role in determining interactions between plasmids and the host chromosome.

Our previous studies revealed that while TurA, TurB, and Pmr bind almost identical regions on the chromosome of strain KT2440 and the pCAR1 plasmid, their regulons differ (14). Analyses of the interactions between the N-terminal domains of TurA, TurB, and Pmr indicated different affinities for hetero-oligomer complex formation among the terminal dimerization sites of the three proteins (13, 15), suggesting that composition of the hetero-oligomers might explain the functional differences between the three MvaT proteins. Four of the five residues forming salt bridges in the TurB_nt_50_ structure are conserved in TurA, and three of these residues are conserved in Pmr (Fig. 4). Of the twelve amino acid residues found to participate in hydrophobic interactions, eight and seven residues are conserved or similar in TurA and Pmr, respectively (Fig. 4). The crystal structure of TurB_nt_50_ could not explain the importance of some amino acid residues (E6, K15, and R20) in the oligomerization of Pmr (Fig. 4). Future detailed analyses of the impact of these differences on the physical interactions among the three MvaT proteins would lead to better understanding of the dynamics of horizontal gene transfer and integration of foreign DNA sequences into the resident transcriptional networks.

### Conclusion

In this study, we characterized the structural basis for oligomerization of MvaT proteins using the native terminal dimerization site of the TurB N-terminal oligomerization site. H-NS family proteins are considered major effectors in the cross-talk between MGE and the host chromosome. Our results provide mechanistic insight into how interactions between MvaT homologs might shape genetic exchanges within microbial communities. Detailed structural studies of the oligomerization manner of other chromosome- and plasmid-borne H-NS family proteins would lead to better understanding of the regulation of transcriptional networks in plasmid-harboring host cells and the overall impact of these mechanisms on microbial evolution.

## MATERIALS AND METHODS

### Protein expression and purification

TurB_nt_50_ containing histidine (His) tag at the C-terminus was expressed and purified as described previously with some modifications (24, 28). *E. coli* BL21 (DE3) (Novagen, San Diego, CA, USA) harboring pET-C-His-turB_nt_50_ (24) was grown in lysogeny broth medium (29) supplemented with 50 μg/ml kanamycin at 30°C until the optical density at 600 nm reached 0.6–0.8. Protein expression was then induced by addition of 0.5 mM isopropyl β-D-thiogalactopyranoside, followed by an additional incubation at 30°C for 6 h. TurB_nt_50_ was purified with a HiTrap Chelating HP column using an ÄKTA FPLC instrument (GE HealthCare, Little Chalfont, UK) as described previously (13, 28). TurB_nt_50_ was eluted using 475 mM imidazole. The elution fractions were subjected to gel-filtration chromatography with a HiLoad 16/60 Superdex 200 prep-grade column (GE HealthCare) using a procedure described previously (28). For crystallization, the fractions containing pure TurB_nt_50_ were concentrated and buffer-exchanged with crystallization buffer (5 mM Tris-HCl [pH 7.5, 4°C], 0.3 M NaCl, 0.4 M non-detergent sulfobetaine 256, 10% glycerol) using Amicon Ultra 3K device (Merck Millipore, Billerica, MA, USA).

### Crystallization, X-ray data collection, and structure determination

Crystallization of TurB_nt_50_ was performed using the hanging-drop vapor-diffusion method at 6°C. 1 μl of freshly purified protein (6 mg/mL) was mixed with an equal volume of precipitant solution composed of 20% (wt/vol) polyethylene glycol (PEG) 3350, 0.1 M 4,2-Hydroxyethyl,1-piperazineethanesulfonic acid (pH 7.6), and 0.2 M ammonium acetate. Crystals were washed in cryoprotectant containing reservoir solution supplemented with 20% (vol/vol) ethylene glycol, picked up by nylon loops, and flash frozen in liquid nitrogen or 100 K nitrogen stream. Diffraction data were collected at beamlines BL-1A and AR-NE3A (Photon Factory, High Energy Accelerator Research Organization, Tsukuba, Japan). XDS (30) was used for indexing and integration calculations of diffraction data, and Aimless (31) was used for merging and scaling of data sets. Prior to S-SAD phasing, three oscillation data sets collected at 2.7000 Å wavelength were merged into a single intensity data set (TurB_nt_50_ S-SAD). Initial phases were acquired through the CRANK2 pipeline (32) implemented in CCP4 program suite (33). Model building and refinement were performed for the data set collected at 1.0000 Å wavelength (TurB_nt_50_) by iteratively using refmac5 (34), phenix.refine (35), and Coot (36). Visualization and molecular analyses were performed using the UCSF CHIMERA package (37). The atomic coordinates of TurB_nt_50_ have been deposited in the World Protein Data Bank (http://www.wwpdb.org/) under the accession code 8H8H.

### SEC-MALS/RI analysis

Multi-angle light scattering (MALS) and differential refractive index (RI) measurements in line with size-exclusion chromatography (SEC) were performed by using an Alliance 2695 high-performance liquid chromatography system (HPLC) equipped with DAWN HELEOS II (Wyatt Technology, Santa Barbara, CA, USA) and a 2414 RI detector (Waters, Milford, MA, USA). TurB_nt_50_ samples (30 μL) were injected into a Superdex 75 Increase 3.2/300 column (GE HealthCare) equilibrated with buffer containing 20 mM Tris-HCl (pH 8.0), 0.5 M NaCl, 10% glycerol, 0.5 M imidazole, and examined under at a flow rate of 0.1 mL/min. The obtained data were analyzed by the ASTRA 6.1 software (Wyatt Technology).

### SEC-SAXS analysis

SEC-SAXS data were collected at BL-10C of the Photon Factory (Tsukuba, Japan) at 293 K. The SEC-SAXS experiments were performed using a HPLC system, Nexera-i (Shimadzu, Kyoto, Japan), connected with a Superdex 75 increase 3.2/300 column (GE HealthCare) equilibrated with buffer containing 20 mM Tris-HCl (pH 8.0), 0.5 M NaCl, 10% glycerol, and 0.5 M imidazole. TurB_nt_50_ sample (80 μl) was loaded into the column, and the flow rate around the elution peak was set to 0.01 mL/min. 303 scattering images were continuously collected by PILATUS3 2M (Dectris, Baden, Switzerland) with 20 s of exposure. The concentration of the eluted solution was derived from the absorbance at 280 nm of the absorption spectra measured simultaneously by a fiber-optic spectrophotometer, QE65pro (Ocean Optics, Orlando, FL, USA) with a SAXS cell. All scattering images were azimuthally averaged to convert the one-dimensional scattering intensity profiles. The 15 images measured before the sample fraction were selected and averaged to create the background profile. This profile was then subtracted from all the profiles. The scattering intensities were calibrated to the absolute scale with a water intensity as a standard. These processes were performed using SAngler (38). The 11 data from the region near the elution peak (frames 134–144) were selected for the structure analysis, and MOLASS (39) calculated the averaged profile from this region. The radius of gyration (*R_g_*), forward scattering intensity (*I*(0)), and molecular weight were calculated by AUTORG (40) and DatMW in the ATSAS program package (41). The theoretical scattering was calculated using CRYSOL (42). Detailed information on SEC-SAXS data collection and analysis is summarized in Table S1, and these data are deposited in the Small Angle Scattering Biological Data Bank (43) under the ID of SASDUP4.

### Molecular modeling

We generated a concatenated TurB model using the crystal structures of TurB_nt_50_ (PDB code: 8H8H) and TurB_nt_61_-R8A (5B52). The procedure is illustrated in Fig. S3. First, the Cα coordinates of residues 32–49 of TurB_nt_50_ Chain A were superimposed onto those of TurB_nt_61_-R8A Chain A (RMSD 0.46 Å) using PyMOL 2.5.0 (44) (Fig. S3A). Next, the atom coordinates of residues 2–32 of TurB_nt_50_ were connected to residues 33–58 of TurB_nt_61_-R8A to construct a concatenated Model 1 (Fig. S3B). The same procedure was performed for the Chain B coordinates of TurB_nt_50_ and TurB_nt_61_-R8A to obtain a concatenated Model 2. Models 1 and 2 formed a dimer through the central dimerization site. Subsequently, the Cα coordinates of residues 2–27 of a copy of Model 1 in complex with Model 2 were superimposed onto the corresponding region of Chain G of TurB_nt_50_ to obtain a model oligomerized at the terminal dimerization site (Fig. S3C). The same operation was performed for Chain F of TurB_nt_50_. We obtained a tetrameric TurB model and the model was subjected to an energy optimization *in vacuo* using AMBER22 (45) with the Amber ff14SB force field (46).

## Data Availability

The crystal structure described in this study is available in the Protein Data Bank under the accession code 8H8H. The SEC-SAXS data have been deposited in the Small Angle Scattering Biological Data Bank under the accession code SASDUP4. Further inquiries regarding the raw data can be directed to the corresponding author upon reasonable request.

## SUPPLEMENTAL MATERIAL

Supplemental material spectrum.00235-26-s0001.pdf Fig. S1 to S3; Table S1.

Movie S1 spectrum.00235-26-s0002.mp4 Oligomerization model of full-length TurB.

Movie S2 spectrum.00235-26-s0003.mp4 Oligomeric structure of the N-terminal oligomerization domain of H-NS from PDB ID 3NR7.

ASM does not own the copyrights to Supplemental Material that may be linked to, or accessed through, an article. The authors have granted ASM a non-exclusive, world-wide license to publish the Supplemental Material files. Please contact the corresponding author directly for reuse.

## References

[1] Dillon SC, Dorman CJ. 2010. Bacterial nucleoid-associated proteins, nucleoid structure and gene expression. Nat Rev Microbiol 8:185–195. doi:10.1038/nrmicro2261 20140026

[2] Dame RT, Rashid F-Z, Grainger DC. 2020. Chromosome organization in bacteria: mechanistic insights into genome structure and function. Nat Rev Genet 21:227–242. doi:10.1038/s41576-019-0185-4 31767998

[3] Gordon BRG, Li Y, Cote A, Weirauch MT, Ding P, Hughes TR, Navarre WW, Xia B, Liu J. 2011. Structural basis for recognition of AT-rich DNA by unrelated xenogeneic silencing proteins. Proc Natl Acad Sci USA 108:10690–10695. doi:10.1073/pnas.1102544108 21673140 PMC3127928

[4] Ding P, McFarland KA, Jin S, Tong G, Duan B, Yang A, Hughes TR, Liu J, Dove SL, Navarre WW, Xia B. 2015. A novel AT-rich DNA recognition mechanism for bacterial xenogeneic silencer MvaT. PLoS Pathog 11:e1004967. doi:10.1371/journal.ppat.1004967 26068099 PMC4466236

[5] Duan B, Ding P, Hughes TR, Navarre WW, Liu J, Xia B. 2018. How bacterial xenogeneic silencer rok distinguishes foreign from self DNA in its resident genome. Nucleic Acids Res 46:10514–10529. doi:10.1093/nar/gky836 30252102 PMC6212790

[6] San Millan A, MacLean RC. 2017. Fitness costs of plasmids: a limit to plasmid transmission. Microbiol Spectr 5. doi:10.1128/microbiolspec.mtbp-0016-2017 PMC11687550 28944751

[7] Vial L, Hommais F. 2020. Plasmid-chromosome cross-talks. Environ Microbiol 22:540–556. doi:10.1111/1462-2920.14880 31782608

[8] Qin L, Erkelens AM, Ben Bdira F, Dame RT. 2019. The architects of bacterial DNA bridges: a structurally and functionally conserved family of proteins. Open Biol 9:190223. doi:10.1098/rsob.190223 31795918 PMC6936261

[9] Takeda T, Yun C-S, Shintani M, Yamane H, Nojiri H. 2011. Distribution of genes encoding nucleoid-associated protein homologs in plasmids. Int J Evol Biol 2011:685015. doi:10.4061/2011/685015 21350637 PMC3042613

[10] Dorman CJ. 2014. H-NS-like nucleoid-associated proteins, mobile genetic elements and horizontal gene transfer in bacteria. Plasmid 75:1–11. doi:10.1016/j.plasmid.2014.06.004 24998344

[11] Shintani M, Suzuki-Minakuchi C, Nojiri H. 2015. Nucleoid-associated proteins encoded on plasmids: occurrence and mode of function. Plasmid 80:32–44. doi:10.1016/j.plasmid.2015.04.008 25952329

[12] Yun C-S, Suzuki C, Naito K, Takeda T, Takahashi Y, Sai F, Terabayashi T, Miyakoshi M, Shintani M, Nishida H, Yamane H, Nojiri H. 2010. Pmr, a histone-like protein H1 (H-NS) family protein encoded by the IncP-7 plasmid pCAR1, is a key global regulator that alters host function. J Bacteriol 192:4720–4731. doi:10.1128/JB.00591-10 20639326 PMC2937398

[13] Suzuki C, Kawazuma K, Horita S, Terada T, Tanokura M, Okada K, Yamane H, Nojiri H. 2014. Oligomerization mechanisms of an H-NS family protein, Pmr, encoded on the plasmid pCAR1 provide a molecular basis for functions of H-NS family members. PLoS One 9:e105656. doi:10.1371/journal.pone.0105656 25137042 PMC4138198

[14] Yun C-S, Takahashi Y, Shintani M, Takeda T, Suzuki-Minakuchi C, Okada K, Yamane H, Nojiri H. 2016. MvaT family proteins encoded on IncP-7 plasmid pCAR1 and the host chromosome regulate the host transcriptome cooperatively but differently. Appl Environ Microbiol 82:832–842. doi:10.1128/AEM.03071-15 26590283 PMC4725287

[15] Sun Z, Vasileva D, Suzuki-Minakuchi C, Okada K, Luo F, Igarashi Y, Nojiri H. 2018. Differential protein-protein binding affinities of H-NS family proteins encoded on the chromosome of Pseudomonas putida KT2440 and IncP-7 plasmid pCAR1. Biosci Biotechnol Biochem 82:1640–1646. doi:10.1080/09168451.2018.1484277 29924693

[16] Ueguchi C, Suzuki T, Yoshida T, Tanaka K, Mizuno T. 1996. Systematic mutational analysis revealing the functional domain organization of Escherichia coli nucleoid protein H-NS. J Mol Biol 263:149–162. doi:10.1006/jmbi.1996.0566 8913298

[17] Badaut C, Williams R, Arluison V, Bouffartigues E, Robert B, Buc H, Rimsky S. 2002. The degree of oligomerization of the H-NS nucleoid structuring protein is related to specific binding to DNA. J Biol Chem 277:41657–41666. doi:10.1074/jbc.M206037200 12200432

[18] Castang S, Dove SL. 2010. High-order oligomerization is required for the function of the H-NS family member MvaT in Pseudomonas aeruginosa. Mol Microbiol 78:916–931. doi:10.1111/j.1365-2958.2010.07378.x 20815825 PMC2978250

[19] Esposito D, Petrovic A, Harris R, Ono S, Eccleston JF, Mbabaali A, Haq I, Higgins CF, Hinton JCD, Driscoll PC, Ladbury JE. 2002. H-NS oligomerization domain structure reveals the mechanism for high order self-association of the intact protein. J Mol Biol 324:841–850. doi:10.1016/s0022-2836(02)01141-5 12460581

[20] Bloch V, Yang Y, Margeat E, Chavanieu A, Augé MT, Robert B, Arold S, Rimsky S, Kochoyan M. 2003. The H-NS dimerization domain defines a new fold contributing to DNA recognition. Nat Struct Biol 10:212–218. doi:10.1038/nsb904 12592399

[21] Cerdan R, Bloch V, Yang Y, Bertin P, Dumas C, Rimsky S, Kochoyan M, Arold ST. 2003. Crystal structure of the N-terminal dimerisation domain of VicH, the H-NS-like protein of Vibrio cholerae. J Mol Biol 334:179–185. doi:10.1016/j.jmb.2003.09.051 14607110

[22] Arold ST, Leonard PG, Parkinson GN, Ladbury JE. 2010. H-NS forms a superhelical protein scaffold for DNA condensation. Proc Natl Acad Sci USA 107:15728–15732. doi:10.1073/pnas.1006966107 20798056 PMC2936596

[23] Summers EL, Meindl K, Usón I, Mitra AK, Radjainia M, Colangeli R, Alland D, Arcus VL. 2012. The structure of the oligomerization domain of Lsr2 from Mycobacterium tuberculosis reveals a mechanism for chromosome organization and protection. PLoS One 7:e38542. doi:10.1371/journal.pone.0038542 22719899 PMC3374832

[24] Suzuki-Minakuchi C, Kawazuma K, Matsuzawa J, Vasileva D, Fujimoto Z, Terada T, Okada K, Nojiri H. 2016. Structural similarities and differences in H-NS family proteins revealed by the N-terminal structure of TurB in Pseudomonas putida KT2440. FEBS Lett 590:3583–3594. doi:10.1002/1873-3468.12425 27709616

[25] Deighan P, Beloin C, Dorman CJ. 2003. Three-way interactions among the Sfh, StpA and H-NS nucleoid-structuring proteins of Shigella flexneri 2a strain 2457T. Mol Microbiol 48:1401–1416. doi:10.1046/j.1365-2958.2003.03515.x 12787365

[26] Dillon SC, Cameron ADS, Hokamp K, Lucchini S, Hinton JCD, Dorman CJ. 2010. Genome-wide analysis of the H-NS and Sfh regulatory networks in Salmonella Typhimurium identifies a plasmid-encoded transcription silencing mechanism. Mol Microbiol 76:1250–1265. doi:10.1111/j.1365-2958.2010.07173.x 20444106

[27] Baehler E, de Werra P, Wick LY, Péchy-Tarr M, Mathys S, Maurhofer M, Keel C. 2006. Two novel MvaT-like global regulators control exoproduct formation and biocontrol activity in root-associated Pseudomonas fluorescens CHA0. Mol Plant Microbe Interact 19:313–329. doi:10.1094/MPMI-19-0313 16570661

[28] Suzuki C, Yun C-S, Umeda T, Terabayashi T, Watanabe K, Yamane H, Nojiri H. 2011. Oligomerization and DNA-binding capacity of Pmr, a histone-like protein H1 (H-NS) family protein encoded on IncP-7 carbazole-degradative plasmid pCAR1. Biosci Biotechnol Biochem 75:711–717. doi:10.1271/bbb.100841 21512245

[29] Sambrook J, Russell DW. 2001. Molecular cloning: a laboratory manual

[30] Kabsch W. 2010. XDS. Acta Crystallogr D Biol Crystallogr 66:125–132. doi:10.1107/S0907444909047337 20124692 PMC2815665

[31] Evans PR, Murshudov GN. 2013. How good are my data and what is the resolution? Acta Crystallogr D Biol Crystallogr 69:1204–1214. doi:10.1107/S0907444913000061 23793146 PMC3689523

[32] Skubák P, Araç D, Bowler MW, Correia AR, Hoelz A, Larsen S, Leonard GA, McCarthy AA, McSweeney S, Mueller-Dieckmann C, Otten H, Salzman G, Pannu NS. 2018. A new MR-SAD algorithm for the automatic building of protein models from low-resolution X-ray data and a poor starting model. IUCrJ 5:166–171. doi:10.1107/S2052252517017961 PMC5947721 29765606

[33] Collaborative Computational Project. 1994. The CCP4 suite: programs for protein crystallography. Acta Crystallogr D Biol Crystallogr 50:760–763. doi:10.1107/S0907444994003112 15299374

[34] Murshudov GN, Skubák P, Lebedev AA, Pannu NS, Steiner RA, Nicholls RA, Winn MD, Long F, Vagin AA. 2011. REFMAC5 for the refinement of macromolecular crystal structures. Acta Crystallogr D Biol Crystallogr 67:355–367. doi:10.1107/S0907444911001314 21460454 PMC3069751

[35] Adams PD, Afonine PV, Bunkóczi G, Chen VB, Davis IW, Echols N, Headd JJ, Hung L-W, Kapral GJ, Grosse-Kunstleve RW, McCoy AJ, Moriarty NW, Oeffner R, Read RJ, Richardson DC, Richardson JS, Terwilliger TC, Zwart PH. 2010. PHENIX: a comprehensive python-based system for macromolecular structure solution. Acta Crystallogr D Biol Crystallogr 66:213–221. doi:10.1107/S0907444909052925 20124702 PMC2815670

[36] Emsley P, Lohkamp B, Scott WG, Cowtan K. 2010. Features and development of Coot. Acta Crystallogr D Biol Crystallogr 66:486–501. doi:10.1107/S0907444910007493 20383002 PMC2852313

[37] Pettersen EF, Goddard TD, Huang CC, Couch GS, Greenblatt DM, Meng EC, Ferrin TE. 2004. UCSF Chimera--a visualization system for exploratory research and analysis. J Comput Chem 25:1605–1612. doi:10.1002/jcc.20084 15264254

[38] Shimizu N, Yatabe K, Nagatani Y, Saijyo S, Kosuge T, Igarashi N. 2016. Software development for analysis of small-angle x-ray scattering data. AIP Conf Proc 1741:050017. doi:10.1063/1.4952937

[39] Yonezawa K, Takahashi M, Yatabe K, Nagatani Y, Shimizu N. 2023. MOLASS: software for automatic processing of matrix data obtained from small-angle X-ray scattering and UV-visible spectroscopy combined with size-exclusion chromatography. Biophys Physicobiol 20:e200001. doi:10.2142/biophysico.bppb-v20.0001 37229310 PMC10203098

[40] Petoukhov MV, Konarev PV, Kikhney AG, Svergun DI. 2007. ATSAS 2.1 – towards automated and web-supported small-angle scattering data analysis. J Appl Crystallogr 40:s223–s228. doi:10.1107/S0021889807002853

[41] Hajizadeh NR, Franke D, Jeffries CM, Svergun DI. 2018. Consensus Bayesian assessment of protein molecular mass from solution X-ray scattering data. Sci Rep 8:7204. doi:10.1038/s41598-018-25355-2 29739979 PMC5940760

[42] Svergun D, Barberato C, Koch MHJ. 1995. CRYSOL – a program to evaluate X-ray solution scattering of biological macromolecules from atomic coordinates. J Appl Crystallogr 28:768–773. doi:10.1107/S0021889895007047

[43] Kikhney AG, Borges CR, Molodenskiy DS, Jeffries CM, Svergun DI. 2020. SASBDB: towards an automatically curated and validated repository for biological scattering data. Protein Sci 29:66–75. doi:10.1002/pro.3731 31576635 PMC6933840

[44] Schrödinger, LLC. The PyMOL Molecular Graphics System, Version 2.5. 2021 Available from: http://www.pymol.org/

[45] Case DA, Metin Aktulga H, Belfon K, Ben-Shalom I, Berryman JT, Andrés Cisneros G, Cruzeiro VWD, Darden TA, Duke RE, Giambasu G, et al.. 2022. Amber 2022. University of California, San Francisco.

[46] Maier JA, Martinez C, Kasavajhala K, Wickstrom L, Hauser KE, Simmerling C. 2015. ff14SB: improving the accuracy of protein side chain and backbone parameters from ff99SB. J Chem Theory Comput 11:3696–3713. doi:10.1021/acs.jctc.5b00255 26574453 PMC4821407

