# Supplementary material for: Structural characterization of the native oligomerization mode of MvaT proteins in Pseudomonas
Source: Microbiol Spectr. 2026 Apr 6;14(5):e00235-26. doi: 10.1128/spectrum.00235-26 (PMC13141940; doi:10.1128/spectrum.00235-26)
Supplement: Supplemental material — Fig. S1 to S3; Table S1. [file spectrum.00235-26-s0001.pdf]

Supplemental Material

Structural characterization of the native oligomerization mode of MvaT proteins in *Pseudomonas*

Delyana Vasileva[a]*, Chiho Suzuki-Minakuchi[a,b #], Takatoshi Arakawa[c,d], Yoshitaka Moriwaki[b,c,e], Kento Yonezawa[f,g], Nobutaka Shimizu[f,h], Zui Fujimoto[i], Tohru Terada[b,c], Kazunori Okada[a], Hideaki Nojiri[a, b #]

[a]Agro-Biotechnology Research Center, Graduate School of Agricultural and Life Sciences, The University of Tokyo, Tokyo, Japan

[b]Collaborative Research Institute for Innovative Microbiology, The University of Tokyo, Tokyo, Japan

[c]Department of Biotechnology, Graduate School of Agricultural and Life Sciences, The University of Tokyo, Tokyo, Japan

[d]Faculty of Pharmaceutical Sciences, Tokyo University of Science, Chiba, Japan

[e]Medical Research Laboratory, Institute of Integrated Research, Institute of Science Tokyo, Tokyo, Japan

[f]Institute of Materials Structure Science, High Energy Accelerator Research Organization (KEK), Ibaraki, Japan

[g]Center for Digital Green-innovation, Nara Institute of Science and Technology, Nara, Japan

[h]Life Science Research Infrastructure Group, R&D of Technology and Systems for Synchrotron Radiation Applications Division, RIKEN SPring-8 Center, Hyogo, Japan

[i]Research Center for Advanced Analysis, National Agriculture and Food Research Organization (NARO), Ibaraki, Japan

[#]Address correspondence to Chiho Suzuki-Minakuchi, csmina@g.ecc.u-tokyo.ac.jp; Hideaki Nojiri, anojiri@g.ecc.u-tokyo.ac.jp.

*Present address: Delyana Vasileva, Oak Ridge National Laboratory, Oak Ridge, Tennessee, USA

The supplemental material includes:

-Fig. S1

-Fig. S2

-Fig. S3

-Movie S1

-Movie S2

-Table S1

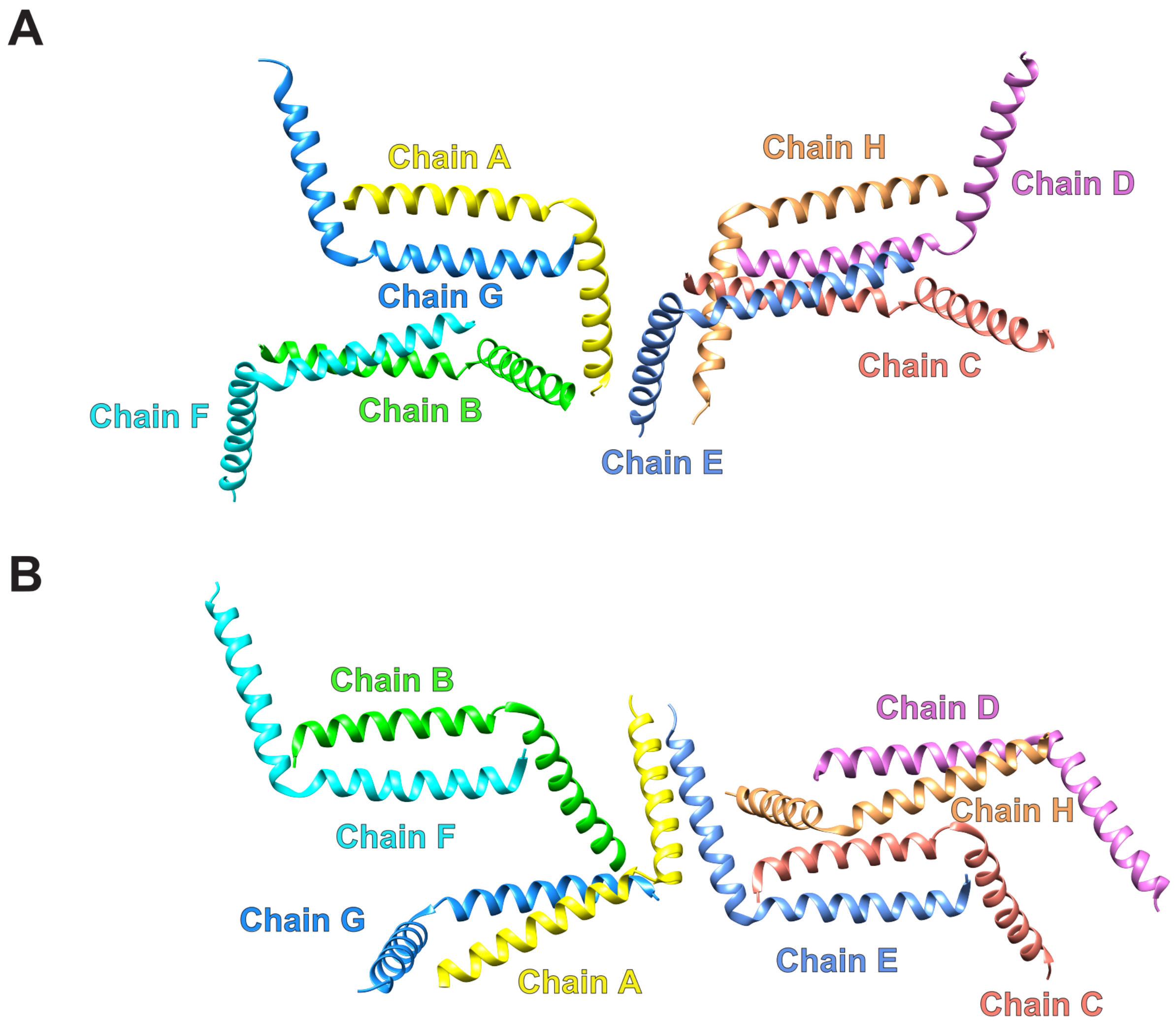

Figure S1. Packing of the eight molecules present in the asymmetric unit of the TurB_nt$_{50}$ crystal. (A) and (B) show the arrangement of the TurB_nt$_{50}$ monomers forming four dimers, as also illustrated in Fig. 1A. A ribbon representation of the eight molecules (Chains A (yellow), B (green), C (salmon), D (pink), E (dark blue), F (cyan), G (blue), H (brown)) is shown.

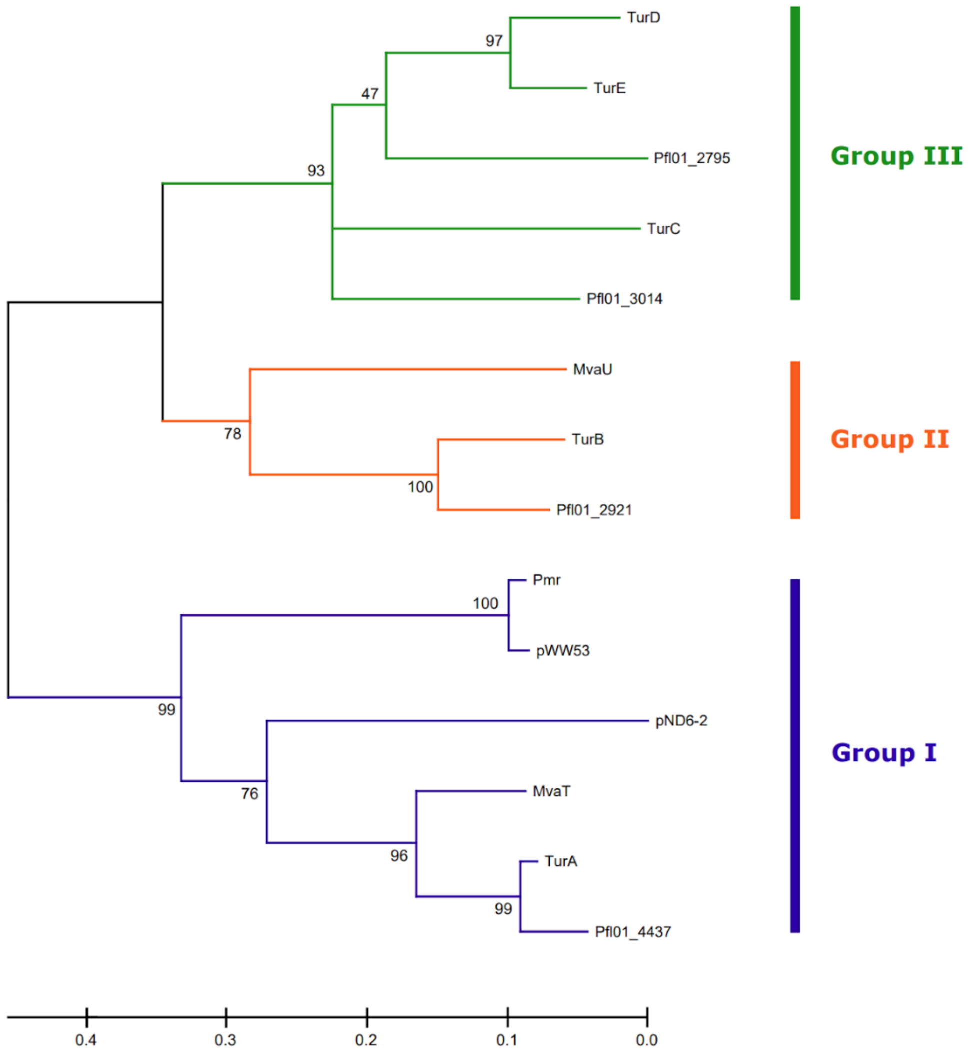

Figure S2. Phylogenetic tree of representative MvaT homologues. The tree was generated using the Neighbor-Joining method (1) with the MEGA11 software (2). Bootstrap values are indicated and correspond to 1000 replications. The scale bar represents the number of substitutions per site. The evolutionary distances were computed using the Jones-Taylor-Thornton matrix-based method (3). Full-length amino acid sequences of TurA (PP_1366), TurB (PP_3765), TurC (PP_0017), TurD (PP_3693) and TurE (PP_2947) of *P. putida* KT2440, MvaT (PA4315) and MvaU (PA2667) of *P. aeruginosa* PAO1, Pfl01_2795, Pfl01_2921, Pfl01_3014 and Pfl01_4437 of *P. fluorescens* Pf0-1, and MvaT proteins encoded on plasmids pCAR1 (Pmr), pWW53 and pND6-2 were used to generate the tree. The amino acid alignment was carried out using ClustalW (4).

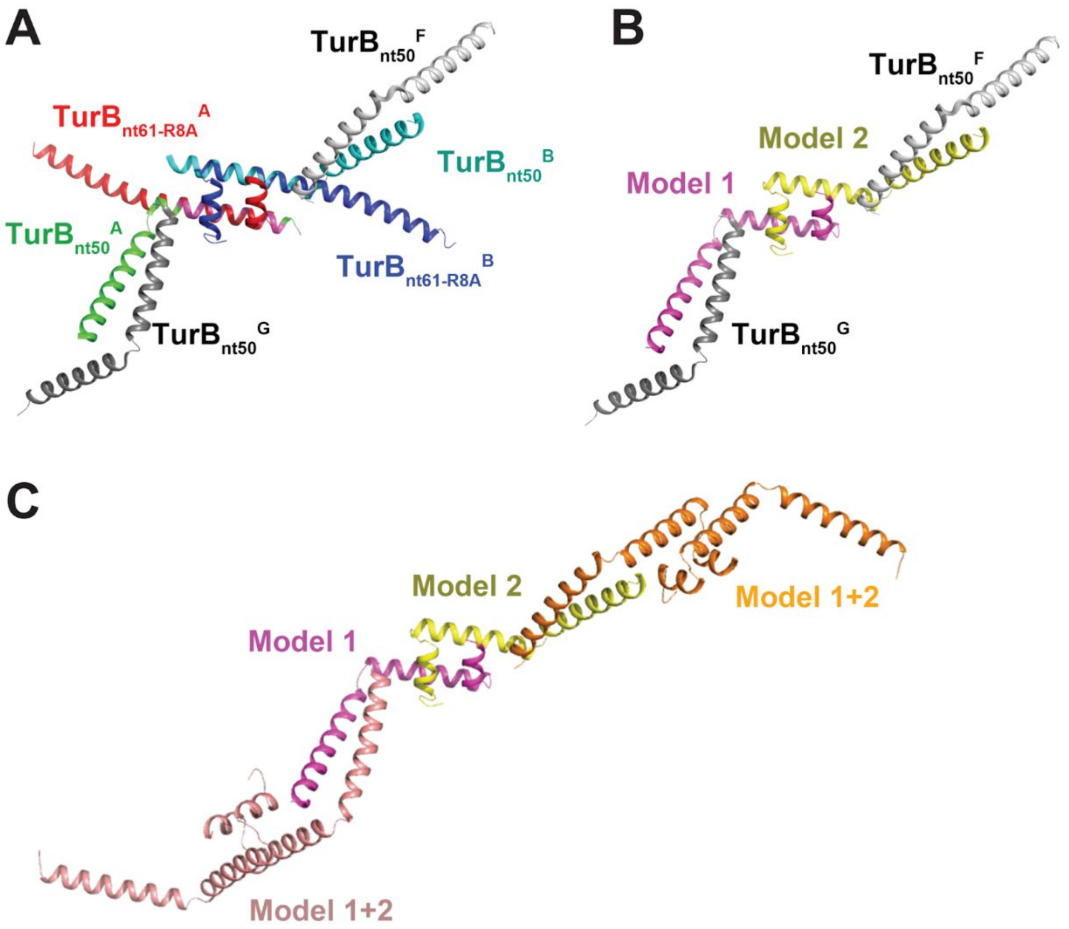

Figure S3. Construction of the oligomeric TurB model. (A) Initial stage. The Cα coordinates of residues 32–49 of chain A of TurB_nt$_{50}$ (green) dimerized with its chain G (gray) was superimposed onto those of TurB_nt$_{61}$-R8A (red). Similarly, another dimer of TurB_nt$_{50}$-Chain B (cyan) and F (white) was superimposed onto the structure of TurB_nt$_{61}$-R8A-Chain B (deep blue). (B) Second stage. Residues 2–32 of TurB_nt$_{50}$ were connected to residues 33–58 of TurB_nt$_{61}$-R8A to build Model 1 (purple) and 2 (yellow). (C) Third stage. Residues 2–27 of a dimer Model 1+2 (pink) were superimposed onto chain G of TurB_nt$_{50}$. The same operation was performed for another copy of Model 1+2 (orange) and Chain F of TurB_nt$_{50}$. Chain F and G were then removed.

Movie S1. **Eight-second movie showing an oligomerization model of full-length TurB.** The model consists of four dimers connected through their terminal dimerization sites. Individual monomers are highlighted in different colors.

Movie S2. Seven-second movie showing the oligomeric structure of the N-terminal oligomerization domain of H-NS from PDB ID 3NR7 (5).

Table S1. Details of small-angle scattering (SAS) data collection and analysis.

**Sample details**	
Organism	*Pseudomonas putida* KT2440
Source	*E. coli* expressed
Uniprot ID	Q88GF9
Extinction coefficient (A_{280nm}, $Abs_{0.1\%}$ (w/v))	0.259
Partial specific volume ($cm^3 g^{-1}$)	0.741
Scattering contrast (cm^{-2})	2.550×10^{10}
MW from chemical composition (Da)	5758.71
Initial concentration for injection (mg ml^{-1})	14
Injection volume (µl)	80
Concentration (mg ml^{-1})	1.411-1.477
Concentration method	UV-Vis spectroscopy
Solvent composition	20 mM Tris-HCl, 0.5 M NaCl, 10% glycerol, 0.5 M imidazole, pH 8.0
**SAS data collection parameters**	
Source, Instrument	Photon Factory, BL-10C
Wavelength (Å)	1.5
Camera length (mm)	1011.7
Beam geometry (µm)	V350 × H550<br>Bent cylindrical mirror + 2 slits + 1 pinhole
Q-measurement range ($Å^{-1}$)	0.010-0.608
Absolute scaling method	Comparison with scattering from pure H_2O
Basis for normalization to constant counts	Normalized to incident intensity by µ ion chamber
Method for monitoring radiation damage	Data frame-by-frame comparison
Exposure time, Number of images	20 sec, 303
Path length	1 mm
Sample temperature (K)	293
**Software used SAS data reduction, analysis and interpretation**	
SAS data processing	*SAngler*, *MOLASS*
Calculation of contrast and PSV values	*MULCh* (6)
Basic analyses: (Guinier, M. W.)	*PRIMUSqt from ATSAS*
Evaluation compared with the crystal structure	*CRYSOL from ATSAS*
**Guinier analysis**	
I(0) (cm^{-1})	0.022 +/- 0.0001
Rg (Å)	28.8 +/- 1.9
Q-range ($Å^{-1}$)	0.0150-0.0449
M from Bayesian interface (ratio to predicted value)<br>[Credibility Interval]	11250 (1.95)<br>[9950-13100]
CRYSOL	
Q-range ($Å^{-1}$) for fitting	0.0135-0.299
R_g (Å) from envelope	24.21
Adjustable parameters in the model fit	Envelope
χ^2 value	8.2

References

1. Saitou N, Nei M. 1987. The neighbor-joining method: a new method for reconstructing phylogenetic trees. Mol Biol Evol 4:406–425.

2. Tamura K, Stecher G, Kumar S. 2021. MEGA11: Molecular Evolutionary Genetics Analysis Version 11. Mol Biol Evol 38:3022–3027.

3. Jones DT, Taylor WR, Thornton JM. 1992. The rapid generation of mutation data matrices from protein sequences. Comput Appl Biosci 8:275–282.

4. Thompson JD, Higgins DG, Gibson TJ. 1994. CLUSTAL W: improving the sensitivity of progressive multiple sequence alignment through sequence weighting, position-specific gap penalties and weight matrix choice. Nucleic Acids Res 22:4673–4680.

5. Arold ST, Leonard PG, Parkinson GN, Ladbury JE. 2010. H-NS forms a superhelical protein scaffold for DNA condensation. Proc Natl Acad Sci U S A 107:15728–15732.

6. Whitten AE, Cai S, Trewhella J. 2008. MULCh: modules for the analysis of small-angle neutron contrast variation data from biomolecular assemblies. J Appl Crystallogr 41:222-226.
